# MiR-520b as a novel molecular target for suppressing stemness phenotype of head-neck cancer by inhibiting CD44

**DOI:** 10.1038/s41598-017-02058-8

**Published:** 2017-05-17

**Authors:** Ya-Ching Lu, Ann-Joy Cheng, Li-Yu Lee, Guo-Rung You, Yan-Liang Li, Hsin-Ying Chen, Joseph T. Chang

**Affiliations:** 1 Department of Medical Biotechnology and Lab Science, College of Medicine, Chang Gung University, Taoyuan, 333 Taiwan; 2 Department of Radiation Oncology, Chang Gung Memorial Hospital - Linko, Taoyuan, 333 Taiwan; 3 Department of Pathology, Chang Gung Memorial Hospital - Linko, Taoyuan, 333 Taiwan; 4 Department of Radiation Oncology, Xiamen Chang Gung Memorial Hospital, Xiamen, Fujian China

## Abstract

Cancer stem cells preferentially acquire the specific characteristics of stress tolerance and high mobility, allowing them to progress to a therapy-refractive state. To identify a critical molecule to regulate cancer stemness is indispensable to erratically cure cancer. In this study, we identified miR-520b as a novel molecular target to suppress head-neck cancer (HNC) with stemness phenotype. MiR-520b inhibited cellular migration and invasion via the mechanism of epithelial-mesenchymal transition. It also sensitized cells to therapeutic drug and irradiation. Significantly, miR-520b suppressed spheroid cell formation, as well as reduced expressions of multiple stemness regulators (Nestin, Twist, Nanog, Oct4). The CD44 molecule was identified as a direct target of miR-520b, as shown by the reverse correlative expressions, the response to miR-520 modulation, the luciferase reporter assay, and the functional rescue analyses. These cellular results were confirmed by a tumor xenograft mice study. Administration of miR-520b dramatically restrained tumorigenesis and liver colonization. Conversely, miR-520b silencing led to an acceleration of tumor growth. Taken together, our study demonstrated that miR-520b inhibits the malignancy of HNC through regulation of cancer stemness conversion by targeting CD44. MiR-520b may serve as an emerging therapeutic target that may be further developed for the intervention of refractory HNC.

## Introduction

Head and neck cancer (HNC) is one of the most prevalent cancers worldwide^1–3^. Despite recent advances in the diagnosis and treatment of HNC, the patient survival rate has not significantly changed due to the development of distant metastases and therapeutic resistance^2–4^. It is therefore essential to investigate the mechanism of this disease more fully and to develop a more effective therapeutic approach.

A model of cancer stem cells has been recently proposed to explain tumor heterogeneity and cancer progression. These cells exhibit both stemness and malignant properties, including self-renewal, high mobility, stress tolerance, and possessing ability to generate various types of progeny cells^5, 6^. Although cancer stem cells represent a small fraction of the overall tumor population, they may be responsible for the ultimate treatment prognosis. It has been hypothesized that current conventional therapies target the rapidly proliferating cells of the tumor bulk, but fail to eradicate the intrinsically resistant type of cancer stem cells. Their self-renewal ability endows these cells with the selective advantage to drive new tumor growth. Thus, targeting to these cells may be an ultimate therapeutic strategy to radically cure cancer^7, 8^. Cancer stem cells have been characterized by specific expression of cell surface markers. CD44 is considered a pan-stemness marker, as highly expression in various types of stem-like carcinomas, including breast, prostate, colorectal and head-neck cancers^9–12^. This molecule may also play critical role in maintaining homeostasis, and serves as an adverse prognostic biomarker^9–12^. However, the regulatory mechanism involved in the CD44 associated cancer stemness is still unclear.

MicroRNAs (miRNAs) are small, non-coding RNA molecules encoded within the genome. A mature miRNA interacts with the 3′ untranslated region (3′-UTR) of its target mRNA, and negatively regulates gene expression through the degradation of the target mRNA to suppress gene translation^13, 14^. It is estimated that approximately half of all human genes are regulated by miRNAs, and each miRNA is predicted to target several hundred transcripts; thus, miRNAs are one of the largest families of gene regulators^13, 14^. Large-scale miRNA screening has been performed and found unique expression profiles in different cancer types^14–17^. MiR-520b belongs to miR-302/372/373/520 family. All miRNAs in this family share similarities in their seed sequences. Recently, the expression of this family of miRNAs has been reported to be altered in several cancers and associated with malignant phenotypes. For example, miR-373 and miR-520c/520 h have been reported with oncogenic roles to promote cell invasion in breast and esophageal cancer cells^18–21^. However, miR-302, miR-372, and miR-520a/520b/520e/520 h have been shown as tumor suppressors to inhibit cell growth or migration in various types of cancers such as breast, liver, and liver^22–29^. This phenomenon implies that miRNA may possess diverse functions in cells dependent on a given regulatory network in a specific tissue type. Nevertheless, the potential role of miR-520 family has not been addressed in HNC. In this study, we examined whether this miRNA family participates in the tumorigenesis of HNC. We determined that miR-520b was a pluripotent tumor suppressor in HNC. The molecular mechanism and potential application of miR-520b were also investigated.

## Results

### Differential expression of miR-302/372/373/520 family members in normal keratinocytes and HNC cell lines

**Figure 1:**
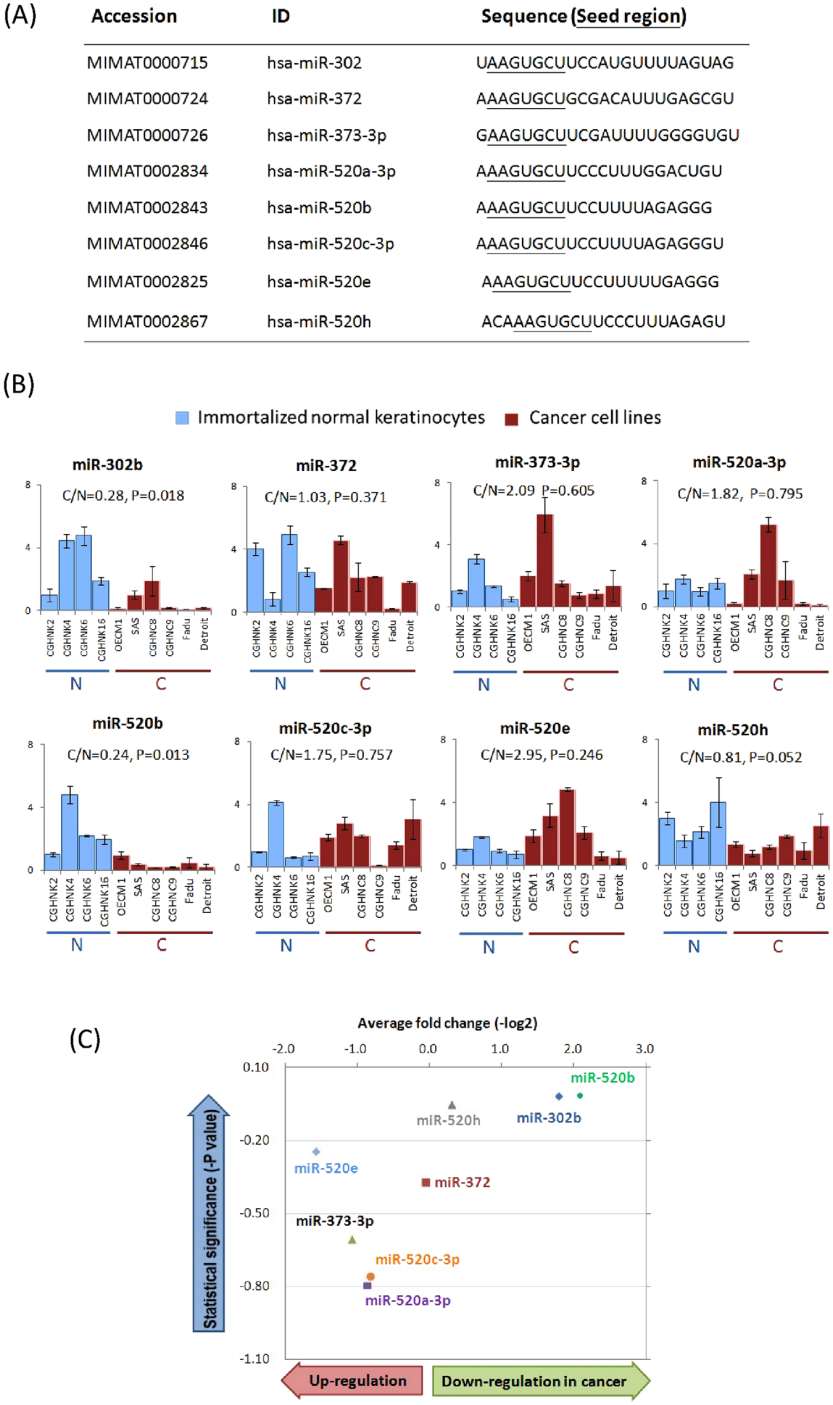
Differential expressions of miR-302/372/373/520 family members between normal keratinocytes and HNC cell lines. (**A**) The miR-302/372/373/520 family members are listed along with the relevant accession number, ID and sequence. Seed regions, which are underlined within the sequence, are the same among all members. (**B**) The relative expression levels of miRNAs of the miR-302/372/373/520 family in HNC cancer cell lines and normal keratinocytes were determined by RT-qPCR. The expression levels as recorded by threshold cycle numbers (Ct) were normalized against an internal control (U6 RNA), and the comparative threshold cycle method (ΔCt) was used to determine the relative miRNA expression. (**C**) The overall view of the differential expression of 8 miRNAs between cancerous and normal samples, with the average fold change (X-axis) and statistical *P* value (Y-axis) for each miRNA (t-test).

To determine the potential role of the miR-302/372/373/520 family in HNC, the expression levels of 8 miRNAs (miR-302b, miR-372, miR-373, miR-520a, miR-520b, miR-520c, miR-520e and miR-520h) were examined in 4 normal keratinocyte cell lines and 6 HNC cell lines. For these miRNAs, the mature sequences with underlined seed regions are listed in Fig. 1A. The relative expression of each miRNA in these cells is shown in Fig. 1B. By using a 1.5-fold average difference between normal and cancer cells as a cut-off point, this family of miRNAs can be categorized into three groups. In general, the expression of miR-373, miR-520a and miR-520e was elevated in cancer cell lines, which indicates oncogenic functions of these miRNAs in HNC. However, miR-302b and miR-520b were down-regulated in cancer cells, indicating they possess tumor suppressive functions in HNC. Nevertheless, miR-372 and miR-520c showed minor changes when compared between normal and cancer cell lines, implying a minimal effect of these miRNAs in HNC. To obtain a more comprehensive view of the expression of these miRNAs, the analysis presented in Fig. 1C shows both the fold-change and statistical *P* values of each miRNA between the two groups (normal and cancer). miR-520b exhibited the highest level of altered expression (by 24% of down-regulation, *P* = 0.013), which indicates a significant role of this miRNA during HNC formation.

### MiR-520b sensitizes cells to chemotherapy drugs and irradiation

**Figure 2:**
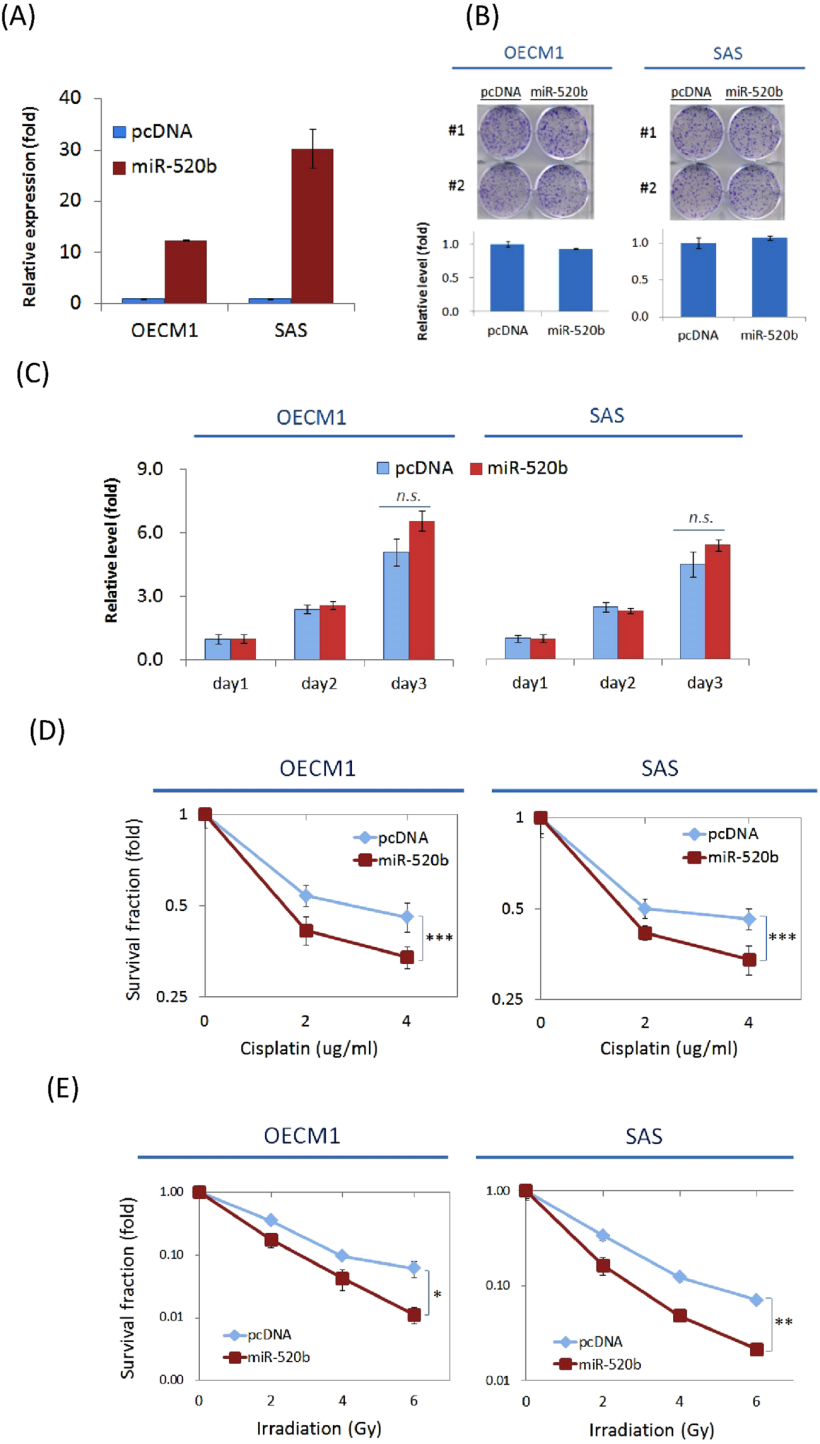
MiR-520b sensitizes cells to chemotherapy drugs and irradiation, while minimal effect on cell growth. (**A**) Cells were transfected with the miR-520b over-expression plasmid (miR-520b) or the vector (pcDNA) and were cultured for 1 day. The miR-520b expression levels were determined. (**B**) The effects of miR-520b modulation on colony formation. A total of 1 × 10^5^ cells were seeded in 6-well plates for 7 days to allow cell colony formation. The number of colonies was then counted. (**C**) The effects of miR-520b modulation on cell growth. A total of 1 × 10^6^ cells were re-seeded in 100-mm plates and the cell numbers were determined daily up to 3 days. (**D**) To detect the chemosensitivity of the cells, the relative number of surviving cells was determined after treatment with various doses of cisplatin (0 to 4 μg/ml) for 2 days. (**E**) To determine radiosensitivity, the colony survival fractions were determined after the cells were irradiated with various doses (0 to 6 Gy). All the experiments were performed duplicate for three times, and the similar results were obtained. The error bars shown in the relevant figures indicated the standard deviation of the three independent experiments. (*p < 0.05, **p < 0.01, ***p < 0.001, *n.s*.: non-significance, *t*-test).

We first determine the potential effects of miR-520b on cell growth and chemo-radiosensitivity. After exogenous transduction of a miR-520b- over-expression plasmid into HNC (OECM1 and SAS) cells, significant up-regulation of this miRNA was observed (Fig. 2A), which indicates successful modulation of miR-520b in HNC cells. The effect on cellular growth was then examined. The over-expression of miR-520b in either OECM1 or SAS cells showed no significant difference in terms of colony formation (Fig. 2B) or cell proliferation (Fig. 2C), which suggests that miR-520b exerts minimal effect on growth regulation.

The potential effects of miR-520b on the sensitivity of cells to cisplatin and radiation were examined. Various concentrations of cisplatin (0–4 μg/ml) were used, and the differential cytotoxicity was examined. As shown in Fig. 2D, transduction of miR-520b increased drug sensitivity in both HNC cell lines. Compared with the controls, cell survival was reduced by 26% and 43% in OECM1 and SAS cells, respectively at 4 μg/ml. Similarly, transduction of miR-520b increased radiosensitivity in both cell lines, as shown by reductions of 82% and 70% in OECM1 and SAS cells at 6 Gy, respectively (Fig. 2E). These results suggest that miR-520b possesses a function in sensitizing cells to chemotherapy drugs and irradiation.

### MiR-520b attenuates cell mobility through suppression of EMT

**Figure 3:**
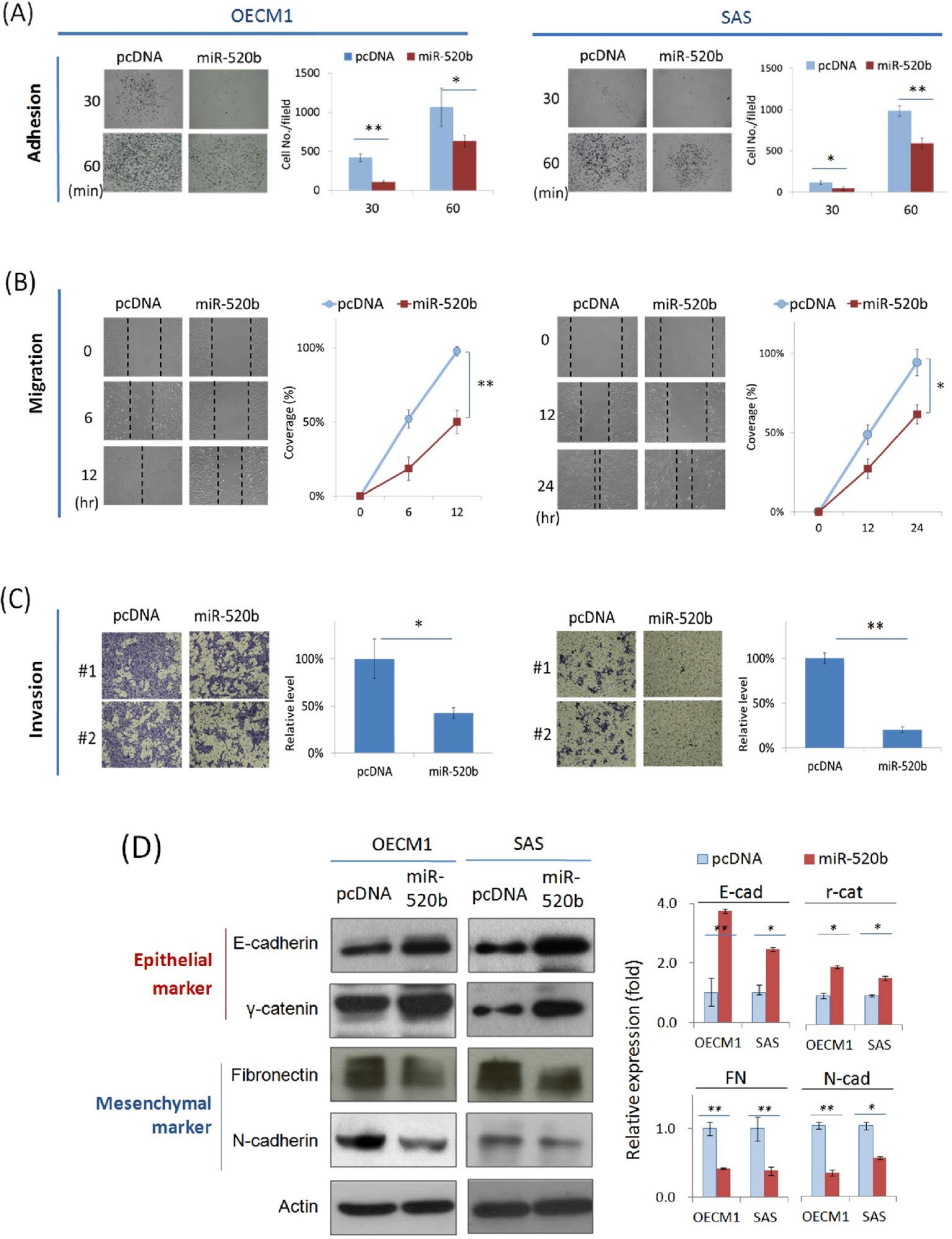
MiR-520b attenuates cell motility and invasion through suppression of EMT. After transfection of miR-520b over-expression plasmids, the HNC cells (OECM1, SAS) were subjected to adhesion, migration and invasion assays as described in the Methods section. (**A**) Mir-520b inhibited cell adhesion. After seeding the miR-520b- or vector- transfected cells into Matrigel coated wells for 30 or 60 mins, the attached cells were stained, photographed and quantified. (**B**) Mir-520b inhibited cell migration. After seeding the miR-520b- or vector- transfected cells into ibidi® culture inserts for 0–12 hrs, cells were subjected to migration analysis as described in the Methods section. Cell migration toward the gap was observed, photographed, and quantified at the indicated times. (**C**) Mir-520b inhibited cell invasion. After seeding the miR-520b- or vector- transfected cells into Matrigel coated membranes for 24 hrs, the cells were subjected to a Matrigel invasion assay as described in the Methods section. The cells that invaded through the Matrigel-coated membranes to the reverse side were stained, photographed, and quantified. (**D**) MiR-520b modulated the expressions of epithelial and mesenchymal marker proteins. After transfection of miR-520b- or the vector plasmids for 24 hrs, cellular proteins were extracted and subjected to western blot analysis for the EMT proteins (E-cadherin, r-catenin, fibronectin, N-cadherin). Actin levels were used as an internal control. The relative density of each sample was determined after normalization to the actin level. All the experiments were performed three times independently and that typical results were shown. The error bars shown in the relevant figures indicated the standard deviation of the quantification results in all experiments. (*p < 0.05, **p < 0.01, t-test).

To test the effect of miR-520b on cell motility, cell adhesion, migration, and invasion were analyzed using solid phase attachment, *in vitro* wound healing, and Matrigel invasion assays. As shown, transduction of miR-520b plasmid significantly inhibited cell adhesion, by decreases of 74% and 61% in OECM1 and SAS cells, respectively after 30 min (Fig. 3A). Consistently, over-expression of miR-520b resulted in a slower migration toward the gap area in both cell lines, with reductions of 49% and 35% in OECM1 and SAS cells at the end of the study period (Fig. 3B). The transduction of cells with miR-520b also substantially reduced cell invasion, with reduction to 47% and 80% in OECM1 and SAS cells (Fig. 3C). Collectively, miR-520b inhibited cell motility through various mechanisms. Because epithelial-mesenchymal transition (EMT) may be acquired to alter cell motility and to increase metastatic potential^30^, we investigated whether miR-520b regulates cell mobility via the suppression of EMT. Epithelial markers (E-cadherin and r-catenin) and mesenchymal markers (Fibronectin and N-cadherin) were examined to determine the status of the cells. Transduction of miR-520b increased the expression level of epithelial-associated molecules, while it decreased the levels of mesenchymal-associated molecules (Fig. 3D). Taken together, these results suggest that miR-520b attenuates cell motility through the suppression of EMT.

### MiR-520b inhibits cancer stemness and modulates pluripotent regulators

**Figure 4:**
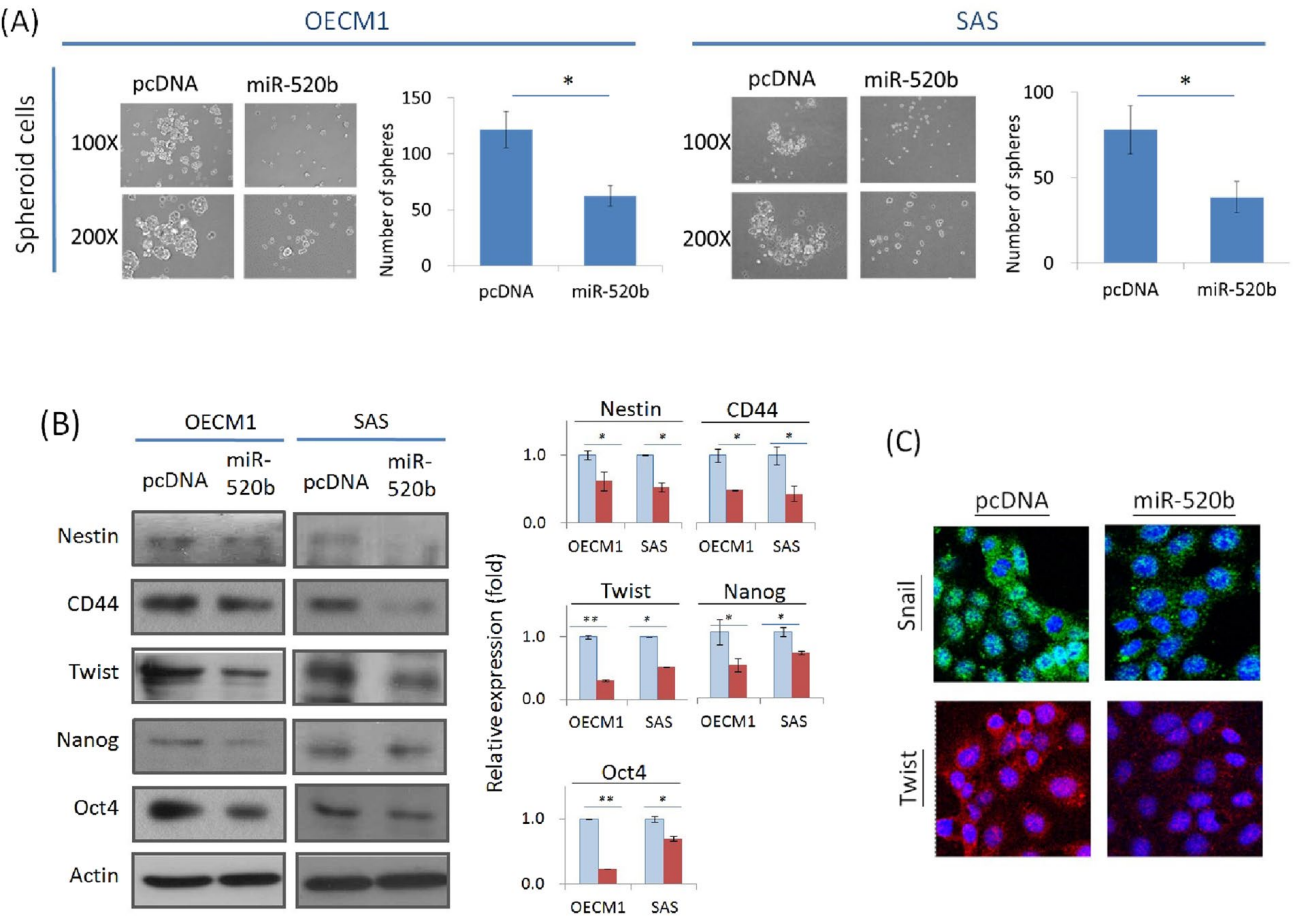
MiR-520b inhibited cancer stemness and modulated pluripotent regulators. (**A**) miR-520b suppressed cell spheroid formation. After the transfection with miR-520b over-expression plasmids, the cells were subjected to sphere formation assays as described in the Methods section. Cell spheres that were grown for 7 days were observed, photographed, and quantified. (**B,C**) miR-520b inhibited the expressions of stemness associated proteins. (**B**) After transfection of miR-520b- or the vector plasmids for 24 hrs, cellular proteins were extracted and subjected to western blot analysis for stemness associated proteins (Nestin, CD44, Twist, Nanog, Oct4). Actin levels were used as an internal control. The relative density of each sample was determined after normalization to the actin level. (**C**) After transfection of miR-520b- or the vector plasmids for 24 hrs, the cells were stained with Snail antibody along with the FITC-conjugated secondary antibody (green imaging), or with Twist antibody along with Cy3-conjugated secondary antibody (red imaging). DAPI staining, as shown in blue, was also performed for nuclear localization. Cell morphology was observed by confocal microscopy. All the experiments were performed three times independently and that typical results were shown. The error bars shown in the relevant figures indicated the standard deviation of the quantification results in all experiments. (*p < 0.05, **p < 0.01, t-test).

Recent studies have shown that CSCs, although they comprise a small subpopulation within a given tumor, are responsible for the initiation of tumorigenesis and that they possess the properties of high invasiveness and chemoradioresistance^5–8^. To determine whether miR-520b also regulates the conversion of cancer stemness, we examined the self-renewal capacity of the cells according to their ability to form spheres, a hallmark of cancer stemness^31, 32^. As shown in Fig. 4A, transduction of cells with miR-520b significantly inhibited the formation of cell spheres, as evidenced by the size and the number (approximately 50% reduction in both OECM1 and SAS cell lines). To extend these results to the molecular level, we determined the expression of several CSC-associated markers (Nestin, CD44) and regulators (Twist, Nanog, Otc 4)^31, 32^. As shown in Fig. 4B, the expression of all of these proteins was significantly inhibited in HNC cell lines upon exogenous transduction of miR-520b; the inhibition ranged from 32% to 85% depending on the specific protein. These results were also confirmed by confocal microscopy examination (Fig. 4C).

### CD44 is a direct regulatory target of miR-520b

**Figure 5:**
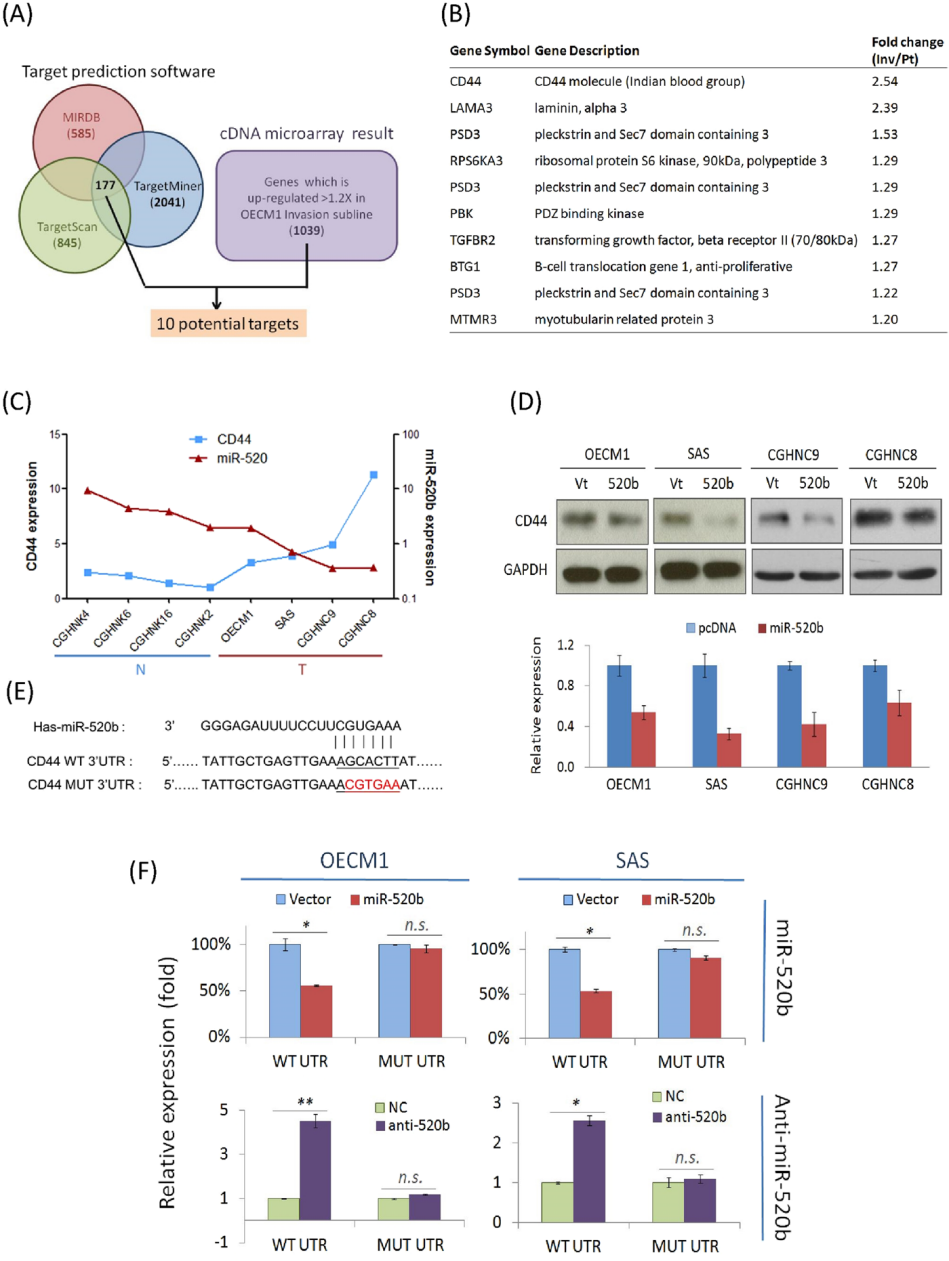
CD44 is a direct regulatory target of miR-520b. (**A**) Bioinformatics-based prediction of the regulatory targets of miR-520b using MIRDB, TargetScan, and TargetMiner computational prediction software. (**B**) List of 10 potential target genes, after integrative analysis of bioinformatic prediction results and the microarray dataset from a highly invasive cell subline. (**C**) The inverse correlation between CD44 and miR-520b expression. Total of 4 lines of normal keratinocytes (CGHNK4, CGHNK6, CGHNK16, CGHNK2) and 4 lines of HNC cells (OECK1, SAS, CGHNC9, CGHNC8) were used. The expression levels of miR-520b were determined by RT-qPCR based assays after normalization to the level of U6 RNA (internal control) for each sample. The CD44 levels were determined by RT-qPCR assays after normalization to GAPDH (internal control) for each sample. (**D**) The effect of miR-520b on CD44 protein expression. After transfection of HNC cells (OECM1, SAS CGHNC8, CGHMC9) with miR-520b over-expression plasmids for 24 hrs, the cellular proteins were extracted and subjected to western blot analysis to determine the CD44 expression. The relative expression was determined after normalization to GAPDH, which was used as an internal control. (**E**) The sequences and binding sites of miR-520b and its target gene, CD44. The mature sequences of miR-520b, as well as the 3′-UTR sequence of the human CD44 gene are shown. The mutant sequence of the 3′-UTR region of CD44 was then designed. (**F**) A luciferase reporter assay was used to determine whether CD44 is a direct target of miR-520b. Cells transfected with miR-520b over-expression plasmids or anti-miR-520b antigomirs were co-transfected with p-WT-UTR or p-MUT-UTR, as indicated in each specific bar of the figure. Renilla luciferase was also co-transfected as a reference control for each condition. Firefly and Renilla luciferase activities were measured by a dual-luciferase reporter assay as described in the Method section. All the experiments were performed duplicate for three times, and the similar results were obtained. The error bars shown in the relevant figures indicated the standard deviation of the three independent experiments. (*p < 0.05, **p < 0.01, n.s.: non-significance, t-test).

In order to identify the potential target of miR-520b, computational prediction software, including MIRDB, TargetMiner and TargetScan were used to predict possible molecules. These programs identified a total of 177 common molecules (Fig. 5A). To narrow the field of prospective candidates, we further analyzed the potential candidates by overlapping this result with that from a microarray dataset of an invasive cell subline^27^. Ten candidates were obtained including CD44, which demonstrated the highest potential (Fig. 5B).

The RT-qPCR assay was first applied to examine the association between miR-520b and CD44 expression in 4 normal keratinocyte cell lines and 4 HNC cell lines. As shown in Fig. 5C, miR-520b was significantly down-regulated in all cancer cell lines, whereas CD44 was up-regulated. The reverse correlation (*r* ^2^ = −0.738, *P* = 0.046) between these two molecules supported the hypothesis that CD44 is a regulatory target of miR-520b. Western blot was then used to determine the potential effect of CD44 protein expression in response to miR-520b modulation. As shown in Fig. 5D, the CD44 protein was significantly down-regulated upon miR-520b transduction, with decreases between 34–67% in the four HNC cell lines.

To validate that CD44 is a regulatory target of miR-520b, a luciferase reporter assay was performed. Reporter plasmids that carry human CD44-3′UTR wide-type sequence (WT) or the mutant sequence (MUT) (Fig. 5E) were co-transfected with either miR-520b expression plasmids or specific antagomirs. The results are shown in Fig. 5F. The over-expression of miR-520b suppressed CD44 WT-UTR reporter activity in both OECM1 and SAS cells (by 44–47%), but had no effect on MUT-UTR activity. Consistently, transduction of cells with miR-520b antagomirs substantially increased CD44 WT-UTR reporter activity in both OECM1 and SAS cells (2.6–4.5-fold), but no effect on the MUT-UTR. Taken together, these results suggest that CD44 is a direct down-stream regulatory target of miR-520b.

### Cellular function of miR-520b occurs through modulation of CD44

**Figure 6:**
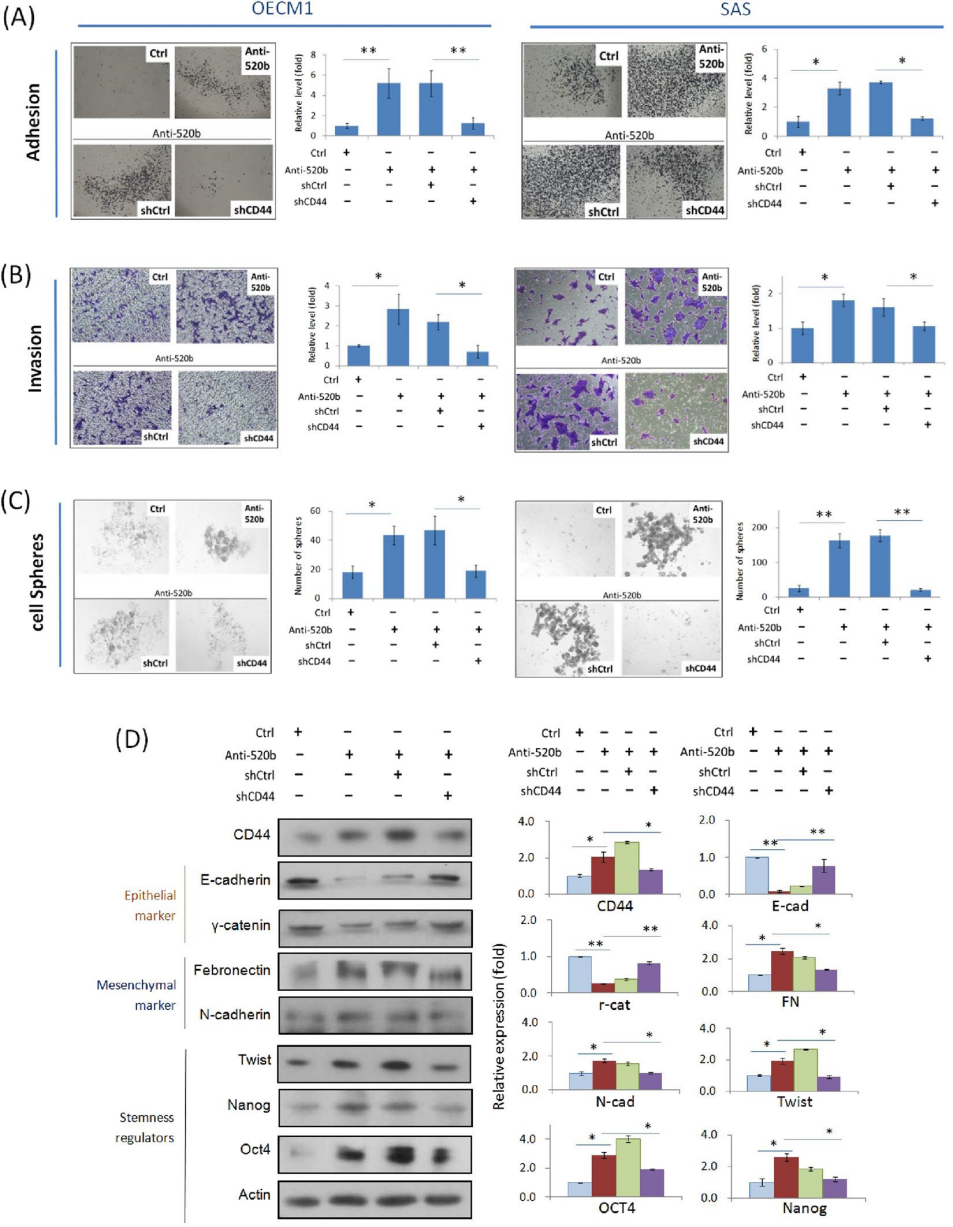
Cellular function of miR-520b occurs through modulation of CD44. Cell adhesion (**A**), invasion (**B**) and sphere formation (**C**) were examined in OECM1 ad SAS cells as described in the Methods section. (**D**) EMT and cancer stemness associated protein expressions were determined by western blot analysis. Actin protein levels were used as an internal control. The relative density of each sample was determined after normalization to the actin level. All the experiments were performed three times independently and that typical results were shown. The error bars shown in the relevant figures indicated the standard deviation of the quantification results in all experiments. (*p < 0.05, **p < 0.01, t-test).

To investigate whether the functions altered by miR-520b may occur via the regulation of CD44, the cellular effects were analyzed when CD44 was silenced in miR-520b-modulated cells. As shown in Fig. 6A, transduction of cells with anti-520b increased cell adhesion (3.6- and 3.3-fold in OECM1 and SAS cells compared to the control); however, this phenomenon was significantly suppressed after the simultaneous inhibition of CD44 (decreased to a level similar to that of the control). Consistently, cell invasion and sphere formation were increased after anti-520b transfection (1.4- to 5.2-fold in two HNC cells compared with the controls); however, these enhancements were abolished upon CD44 silencing (return to levels similar to those of the controls) (Fig. 6B,C). Hence, the cellular effect of miR-520b is apparently CD44-dependent.

To investigate whether EMT- and cancer stemness- associated proteins were also regulated by this miR-520b-CD44 molecular axis, we examined the expression levels of these molecules in response to miR-520b and CD44 co-modulations. The results are shown in Fig. 6D. Transduction of cells with anti-520b substantially inhibited the expression of epithelial markers (E-cadherin and r-catenin), while it increased the levels of mesenchymal markers (Fibronectin and N-cadherin) and stemness regulators (Twist, Nanog, and Oct4). Nevertheless, these molecular alterations were restored upon CD44 suppression. Taking together, miR-520b functions in HNC through the targeted suppression of CD44, which further leads to modulation of EMT- and stemness- associated protein expressions.

### **MiR-520b suppresses tumorigenesis and metastasis*in vivo***

**Figure 7:**
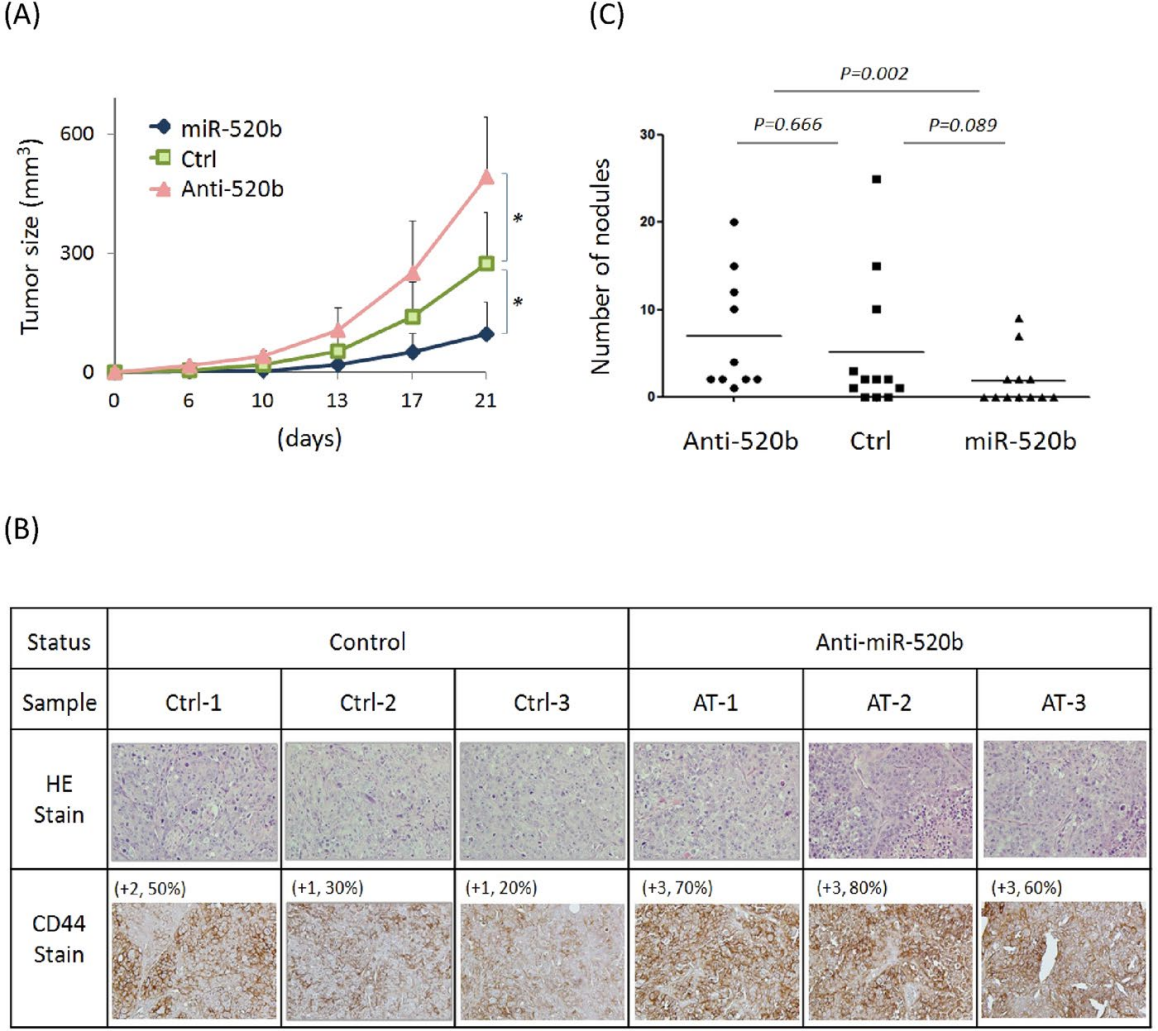
MiR-520b suppresses tumorigenesis and metastasis *in vivo*. (**A**) Total of 1 × 10^6^ Fadu cells were injected subcutaneously into the upper hind limb area (2 sites in left and right side each) of the mice for 1 week. After 6 days, the oligonucleotides of the miR-520 mimic (miR-520b), the controls (Ctrl), or the miR-520b-specific antagomirs (Anti-520b) were administered intravenously into six mice (12 sites) per treatment group, following a boost with anti-miR-520b, control oligonucleotide and a miR-520b mimic twice a week. In each group, the tumor volumes were recorded for up to 21 days, as described in the Method section. The error bars of each data points indicate the positive standard deviations of tumor volumes. Mann-Whitney U test was used for statistical calculation. (*p < 0.05). (**B**) After 21 days of grafting, tumors were removed and subjected to immunohistochemistry (IHC) analysis of CD44 (radish color). The haematoxylin and eosin stains (HE stain) were also performed as controls. Three tumor sections from the groups of anti-miR-520b and control were shown for examples. For each tissue, the intensity score (0 = negative; 1 = weak; 2 = intermediate; 3 = strong) and the fraction score (percentage of positive tumor cells; range = 0–100) of CD44 staining were indicated at the upper left side. (**C**) Total of 5 × 10^5^ FaDu cells were injected into the blood stream via the tail vein. After 6 days, the oligonucleotides of the miR-520 mimic (miR-520b), the controls (Ctrl), or the miR-520b-specific antagomirs (Anti-520b) were administered intravenously into 12 mice per each specific group, following a boost with anti-miR-520b, control oligonucleotide and a miR-520b mimic twice a week. After 21 days, the mice were euthanized and the liver autopsy nodules were examined. The numbers of nodules in the three groups are illustrated. Fisher’s exact test was used for statistical calculation.

To determine the *in vivo* function of miR-520b in HNC, xenografted tumors in BALB/C mice were established. For the determination of the effect of miR-520b on tumor growth, HNC cells were injected subcutaneously into the upper area of the hind limb. After 6 days, the oligonucleotides of the miR-520, the controls, or the miR-520b-specific antagomirs (anti-520b) were administered intravenously into each specific group of mice. Figure 7A shows the average tumor volume between these groups over 21 days of the study. Compared to the control group, tumors in the anti-520b treatment group experienced significant growth and had increased 1.8-fold by day 21. In contrast, tumors in mice given miR-520b mimic oligonucleotides were significantly restrained. We further determined the potential impact of anti-520b treatment on CD44 gene expression in xenograft tumors by immunohistochemistry method. As shown in the Fig. 7B, the anti-520b tumors showed a generally higher level of CD44 expression compared to the controls, in terms of staining density and fraction in tumors. These results suggest that miR-520b possesses an inhibitory effect on tumorigenesis via modulation of CD44 expression.

To determine the effect of miR-520b on tumor metastasis, HNC cells were injected into the blood stream via the tail vein. After 6 days, the miR-520 oligonucleotides, the controls, or the specific antagomirs were administered intravenously into each specific group of mice. After 21 days, the mice were euthanized and the liver autopsy nodules were examined. Figure 7C shows the numbers of liver nodules in each group. In the control group, a wide range (0–25) of nodular colonization was found among different mice. For the anti-520b treatment group, there was no significant difference of tumor colonization compared to the control group. However, the number of nodules was significantly reduced in the miR-520b treatment group, by 64% reduction in average. These results indicate that miR-520b possesses the potential to suppress metastasis.

To approach the clinical significance of miR-520b and CD44 in cancer, we performed prognostic analyses using datasets available online. Result showed that high level of CD44 was associated with shorter relapse-specific survival in HNC (HR = 1.25, *p* = 0.021) (Figure S1) (PrognoScan tool with dataset GSE2837). Conversely, lower level of miR-520b was associated with shorter overall survival in breast cancer (HR = 0.55, *P* = 0.023) (Figure S2) (Kaplan-Meier Plotter with TCGA dataset). These results support our findings that lost miR-520b or elevated CD44 expression may lead to adverse treatment outcome in cancer patients.

## Discussion

Members of a microRNA family, which is defined by the grouping of miRNAs that share a conserved seed region, are expected to regulate the same target gene and to possess a similar function. However, it has been found that these miR-302/520 family members play diverse roles in different cancers^18–29^. Our findings in regards to this family in HNC were partly in agreement with the results of other studies. For example, miR-373 is more highly expressed in HNC cells in general (Fig. 1) and it has been found to promote cell proliferation or invasion in esophageal and breast cancers^18, 19^. The role of miR-302b, which was shown to be significantly reduced in HNC cells (Fig. 1), is consistent with its tumor suppressive function in esophageal and endometrial cancers^22, 28^. MiR-520b and miR-520h, which are both down-regulated in HNC cell lines (Fig. 1), have consistently been found to suppress cell growth and motility in breast, and liver cancers^25, 26^. Apparently, the distinct sequence of a miRNA is a critical parameter for its tissue-specific functions.

The dysregulation of miRNAs may contribute to cancer development. Unlike the function of miR-520b in hepatomas^26^, no significant effect has been observed on cell growth in HNC (Fig. 2B-C). However, transduction of cells with miR-520b sensitizes HNC cells to radiation and drug treatment (Fig. 2D,E). These results are consistent with the findings in regards to miR-520h, which targets the ABCG2 molecule leading to increase drug sensitivity in pancreatic cancer cells^29^. We also found that miR-520b suppresses cell motility in HNC through several mechanisms including cell adhesion, migration and invasion (Fig. 3A–C). These results are consistent with those of previous reports of the inhibition of cell migration in breast cancer^25^. More notably, miR-520b exhibits a strong ability to reduce cancer stemness in HNC (Fig. 4A). This result is supported by the role of the family member miR-302, which inhibits tumorigenicity of human pluripotent stem cells and regulates somatic cell reprogramming^33, 34^. Overall, our study demonstrates that miR-520b regulates multiple malignant properties, including sensitizing cells to chemo-radiotherapy, suppressing cell motility, and attenuating the conversion of cancer stemness. Furthermore, miR-520b achieves these functions may through EMT-associated pathways (Fig. 3D,E) and via pluripotent stemness regulators (Fig. 4B,C).

The function of miRNAs as negative gene regulators in cells is based on their target transcripts. Through an algorithm-based analysis of predicted miRNA targets, plenty of miRNA-gene relationships have been presumed^35^. Distinct databases predict targets based on different computational algorithms. To identify prospective targets of miR-520b, we combined an analysis of three types of miRNA prediction software and a transcriptomic microarray dataset to assess promising candidates (Fig. 5A). Finally, CD44 showed the highest potential among all possible molecules (Fig. 5B). We further confirmed that CD44 was a downstream direct target of miR-520b, as shown by its correlative inverse expression in HNC cells, modulated expression after miR-520b transduction, and by luciferase reporter assays (Fig. 5C–F). Nevertheless, additional target molecules were also reported by other investigators. These include HBXIP and IL8 in breast cancer^25^, and MEKK2 and cyclin D1 in hepatomas^26^. These findings were explained by the hypothesis that a single miRNA is able to regulate several transcripts, which allows for numerous functions of a given miRNA in diverse tissues. It appears that miRNAs cooperate with downstream molecules in specific pathways to maintain cellular homeostasis.

CD44, a cell-surface glycoprotein molecule, is widely expressed in the cell membrane where it contributes to cell adhesion, motility and trafficking. This molecule is also considered a stemness marker in a wide range of tissue types, such as breast, prostate, colorectal and head-neck cancers^9–11^. Aside from a marker protein, in this study, we demonstrated that CD44 plays an active role in several malignant phenotypes, including chemo-radiosensitivity, cell mobility, and self-renewal (Fig. 6A–C). We also confirmed that CD44 acts as a signaling molecule to induce downstream molecular cascades to facilitate EMT and stemness conversion (Fig. 6D). Nevertheless, these malignant phenotypes were reversed upon mir-520b administration. Apparently, CD44 may be a crucial molecule contributing to refractive cancer. It may also act as a signaling molecule that conveys messages from miR-520b to induce downstream molecular cascades leading to tumorigenesis and stemness conversion. Targeting to CD44 via miR-520b may be an effective modality to eradicate HNC with stemness phenotype.

In this study, we used OECM1 and SAS cells throughout all cellular and molecular experiments. There was no significant difference of miR-520b effects on these two cell lines in either cellular functions or molecular targeting (Figs 2 to 6). The OECM1 and SAS cell lines are HPV-negative and -positive, respectively (reference 40). Therefore, miR-520b regulatory mechanism may be HPV- independent in HNC cells.

We further showed that the intravenous administration of miR-520b oligonucleotides significantly inhibited tumorigenesis and liver colonization (Fig. 7A,B). These results indicate that miR-520b possesses a high potential to suppress tumorigenesis and metastasis. Recently, miRNAs have been thought of not only as drug targets but also as therapeutic modalities. The advantages of the application of oligonucleotides as therapeutic agents include their high specificity, easy synthesis or modification, low toxicity and good stability^36^. Despite the challenges presented to deliver these oligonucleotides, miRNA-based therapies have been demonstrated to be feasible and satisfactory in terms of efficacy in various disease models^37, 38^. This promising avenue of therapy is further supported by the fact that a miRNA-based therapeutic has already entered the clinical trial stage^39^. In our study, miR-520b was demonstrated to be a potent agent that functions to attenuate tumorigenesis and metastasis *in vivo*. We concluded that miR-520b inhibits malignancy of HNC through the regulation of cancer stemness by targeting CD44. MiR-520b may serve as an emerging therapeutic modality that can be further developed for intervention in refractory HNC.

## Methods

### Cell lines

Six head and neck cancer cell lines (OECM1, SAS, CGHNC8, CGHNC9, Fadu, and Detroit) and four HPV-immortalized normal keratinocyte cell lines (CGHNK2, CGHNK4, CGHNK6 and CGHNK16) were used in this study^31, 40^. The normal keratinocytes were maintained in KSFM medium with supplements (EGF, Human Recombinant/Bovine Pituitary Extract) (Life Technologies, Inc., Gibco BRL, Rockville, MD, USA). The cancer cell lines were grown in 100% DMEM or RPMI 1640 medium supplemented with 10% fetal bovine serum (Life Technologies, Inc.). All cells were cultured at 37 °C in a humidified atmosphere with 5% CO_2_.

### Cloning and transfection of miR-520b-specific plasmids, CD44-shRNA and inhibitory antagomir oligonucleotides

The stem-loop oligonucleotide of miR-520b (5′-CCCTC TACAG GGAAG CGCTT TCTGT TGTCT GAAAG AAAAG AAAGT GCTTC CTTTT AGAGG G-3′) was inserted into the multiple cloning site of the pcDNA 3.1(+) expression vector to generate the miR-520b expression plasmid, similarly to what has been previously described^41^. The stem-loop shRNA oligonucleotide of the complementary sequence to CD44 (5′-AACTC TGGAC GTCCA TACCC GAAGC TTGGG GTATG GACGT CCAGA GTC-3′) was designed similarly to what has been previously described^42^ and was inserted into the pTOPO-U6 vector to generate the CD44-shRNA plasmids. The miR-520b antagomir and mimic oligonucleotides were purchased from Biotools (New Taipei City, Taiwan). Transfection was performed with Lipofectamine 2000™ reagent (Invitrogen, Grand Island, NY, USA) in OPTI-MEM medium (Invitrogen), and the cells were incubated for 24 h at 37 °C in a humidified atmosphere with 5% CO_2_. Afterward, the medium was replaced with fresh complete medium, and the cells were continuously cultured.

### Cell migration assay

Cell migration was determined by an *in vitro* wound healing assay as previously described^43^. After transfection with the miR-520b expression plasmids or the antagomir oligonucleotides, 70 μl of 4 × 10^5^/ml cells were seeded in ibidi^®^ culture inserts (ibidi LLC, Verona, WI, USA), which were placed inside a 6-well plate. After 8 h of incubation, the culture inserts were detached to form a cell-free gap in the cell monolayer. After the culture medium was changed to medium supplemented with 1% FCS, cell migration toward the gap area was photographed every 6 h.

### Cell invasion assay

Cell invasion was determined using a Matrigel invasion assay as previously described^43^. Briefly, the membrane of the Millicell upper chamber (Millipore, Billerica, MA, USA) was coated with Matrigel (Becton Dickinson Biosciences, Franklin Lakes, NJ, USA) for 12 h at 37 °C. After the transfection of the cells with the miR-520b expression plasmids or the antagomir oligonucleotides, the cells were seeded in the upper chamber with 1% FBS medium. The lower chamber contained complete culture medium (containing 10% FBS) to trap invading cells. After incubation at 37 °C, the cells that invaded through the Matrigel-coated membranes to the reverse side were stained with 0.5% crystal violet and photographed.

### Cell adhesion assay

The cell adhesion assay was performed by a solid phase attachment method similar to what was previously described^44^. Briefly, the 96-well plate was first coated with 30 μl of 200 μg/ml Matrigel per well. After the transfection of cells with the miR-520b expression plasmids or the antagomir oligonucleotides, 5 × 10^5^ cells that were suspended in 1% BSA medium were placed into each well. After incubation for 30/60 min, the non-adherent cells were removed, and the attached cells were fixed in 4% paraformaldehyde, stained with 0.5% crystal violet and photographed.

### Cell sphere formation assay

The sphere formation assay was performed to determine the cell renewal ability as previously described^31^. Briefly, the cells were first cultured in DMEM/F12 medium supplemented with 10 ng/ml of EGF, 10 ng/mL of bFGF and 1X N2 supplement (Invitrogen, Carlsbad, CA, USA). The cells were then diluted as a suspension (1,000 cells/ml), and 1 ml was plated in each well of a 24-well plate, which was pre-coated with 0.6% agarose in 0.3 ml DMEM/F12 medium. After 10 days, the cell spheres were visualized and enumerated.

### Determination of chemosensitivity and radiosensitivity

Radiosensitivity and chemosensitivity were determined by clonogenic survival and MTS survival assays, respectively^45^. After transfection of either the miR-520b expression plasmids or the vectors, 1 × 10^5^ cells were seeded in six-well plates for 8 h. To measure chemosensitivity, the cells were treated with various concentrations (0–4 μg/ml) of cisplatin and allowed to remain in culture for 2 days. The number of surviving cells was counted and compared with the number of untreated surviving cells. To assess radiosensitivity, 1 × 10^3^ cells transfected with either the miR-520b expression plasmids or the vectors (pcDNA) were seeded into 30-mm culture dishes. After an 8-h incubation, the cells were exposed to a range of radiation doses (0–6 Gy) and were continuously cultured for 7 days, at which point the number of surviving colonies was calculated.

### Reverse transcription-quantitative PCR (RT-qPCR) for miR-520 and CD44 molecules

The RNA extraction and RT-qPCR analysis were performed similarly as previously described^46^. Briefly, Total RNA was isolated from cells using TRIzol reagent (Gibco BRL). The PCR primers used for CD44 determination were forward: 5-AGATC AGTCA CAGAC CTGCC-3′, and reverse: 5′-GCAAA CTGCA AGAAT CAAAG CC-3′. The specific TaqMan RT-qPCR assay kits for determination of miR-520b and U6-RNA were purchased from ABI company (Assay ID: 001116 for has-miR-520b and 001093 for RNA-U6, ABI, Forest City, CA, USA). The results of the real-time PCR, which were recorded as threshold cycle numbers, were normalized against an internal control (U6 RNA). The comparative threshold cycle method was used to determine miRNA expression as previously described^46^.

### Protein extraction and western blot analysis

Protein extraction and western blot analysis were performed as previously described^47^. Briefly, cellular proteins were extracted, separated by SDS-PAGE, and transferred to a nitrocellulose membrane. The membrane was hybridized to primary antibodies and then incubated with horseradish peroxidase-conjugated secondary antibodies. The membranes were developed using an ECL developing solution (Millipore, Darmstadt, Germany) followed by autoradiography. The primary antibodies used in this study are listed in Supplementary Table 1.

### Luciferase reporter assay for the CD44-3′UTR

The luciferase reporter assay for the examination of miRNA target genes was performed as previously described^41^. The pMIR-REPORT firefly luciferase vector plasmid (pMIR, Ambion, Grand Island, NY, USA) was used in this experiment. The 3′-UTR region of the wild-type CD44 sequence (NM_000610, CD44 mRNA nt: 4525-5731, length 1,209 bp) was amplified by PCR and cloned downstream of the luciferase vector (p-WT-UTR). A mutant sequence of the miR-520b binding region was also cloned as a validation plasmid (p-MUT-UTR). The p-WT-UTR and p-MUT-UTR plasmids were co-transfected with the miR-520b–specific antagomirs or with the expression plasmids into OECM1 or SAS cells. The pRL-TK vector (Promega, Madison, Wisconsin, USA) containing *Renilla* luciferase was also transfected in each experiment as a reference control. The Firefly and *Renilla* luciferase activities were measured using the Dual-Luciferase Reporter Assay System (Promega) according to the manufacturer’s instructions.

### Immunofluorescence staining and confocal microscopy

Immunofluorescence staining and confocal microscopy were performed as previously described^43^. Briefly, cells were seeded onto coverslips coated with poly-l-lysine and incubated overnight at 37 °C. After the cells were washed, they were fixed in formaldehyde, permeabilized, and blocked with 1% FBS. After an overnight incubation with the primary antibodies, the coverslips were incubated with fluorescence- conjugated secondary antibodies (Molecular Probes, Invitrogen, Carlsbad, CA, USA). The coverslips were then mounted with mounting medium containing DAPI dye (Vector Laboratories, Burlingame, CA, USA), and the fluorescence was visualized using a confocal laser microscope (Leica TCS Sp2 MP, Leica Microsystems, Wetzlar, Germany).

### Experimental animal model

All animal procedures were approved by the IACUC (Institutional Animal Care and Use Committee) at Chang Gung University and followed the guidelines of our institution’s research council for the care and use of laboratory animals. The approval No. is CGU-14-030. The xenografted tumors in mice were established as previously described^43^. Briefly, the immunodeficient mice (BALB/cAnN.Cg-Foxn1nu/CrlNarl, 4 to 5 weeks old) were maintained under standard conditions according to institutional guidelines of animal management. In all, 1 × 10^6^ FaDu cells, which were transfected with 350 pmol of control oligonucleotides (Ctrl), miR-520b antagomir (anti-520b) or miR-520b mimic oligonucleotides, were subcutaneously injected into BALB/c mice. After 6 days, the mice were boosted with 20 ng of each specific oligonucleotide via tail vein injection twice a week. The tumor volume was calculated as length × width × height and was measured every three days. For the metastasis experiment, 5 × 10^5^ FaDu cells, which were transduced with 350 pmol of control oligonucleotide (Ctrl), miR-520b antagomir (anti-520b) or miR-520b mimic oligonucleotides, were injected via the tail vein. Then, the mice were boosted with 20 ng of each specific oligonucleotide via tail vein injection twice a week. After 21 days, the mice were euthanized and the liver autopsy nodules were examined.

## Electronic supplementary material

Supplementary Information

## References

[1] Saman DM (2012). A review of the epidemiology of oral and pharyngeal carcinoma: update. Head Neck Oncol.

[2] Warnakulasuriya S (2009). Global epidemiology of oral and oropharyngeal cancer. Oral Oncol.

[3] Chen YJ (2008). Head and neck cancer in the betel quid chewing area: recent advances in molecular carcinogenesis. Cancer Sci.

[4] Huang Q (2012). Association of HPV infections with second primary tumors in early-staged oral cavity cancer. Oral Dis.

[5] Kreso A, Dick JE (2014). Evolution of the cancer stem cell model. Cell Stem Cell.

[6] Cabrera MC, Hollingsworth RE, Hurt EM (2015). Cancer stem cell plasticity and tumor hierarchy. World J Stem Cells.

[7] Medema JP (2013). Cancer stem cells: the challenges ahead. Nat Cell Biol.

[8] Yang C, Jin K, Tong Y, Cho WC (2015). Therapeutic potential of cancer stem cells. Med Oncol.

[9] Chanmee T, Ontong P, Kimata K, Itano N (2015). Key Roles of Hyaluronan and Its CD44 Receptor in the Stemness and Survival of Cancer Stem Cells. Front Oncol.

[10] Yan Y, Zuo X, Wei D (2015). Concise Review: Emerging Role of CD44 in Cancer Stem Cells: A Promising Biomarker and Therapeutic Target. Stem Cells Transl Med.

[11] Orian-Rousseau V (2015). CD44 Acts as a Signaling Platform Controlling Tumor Progression and Metastasis. Front Immunol.

[12] Horta S (2015). Looking out for cancer stem cells’ properties: the value-driving role of CD44 for personalized medicine. Curr Cancer Drug Targets.

[13] Ha M, Kim VN (2014). Regulation of microRNA biogenesis. Nat Rev.

[14] Jansson MD, Lund AH (2012). MicroRNA and cancer. Mol Oncol.

[15] Bouyssou JM, Manier S, Huynh D, Issa S, Roccaro AM, Ghobrial IM (2014). Regulation of microRNAs in cancer metastasis. Biochim Biophys Acta.

[16] Hayes J, Peruzzi PP, Lawler S (2014). MicroRNAs in cancer: biomarkers, functions and therapy. Trends Mol Med.

[17] Lu YC (2016). MiR-196, an emerging cancer biomarker for digestive tract cancers. J Cancer.

[18] Lee KH (2009). MicroRNA-373 (miR-373) post-transcriptionally regulates large tumor suppressor, homolog 2 (LATS2) and stimulates proliferation in human esophageal cancer. Exp Cell Res.

[19] Huang Q (2008). The microRNAs miR-373 and miR-520c promote tumor invasion and metastasis. Nat Cell Biol.

[20] Su JL (2010). Downregulation of microRNA miR-520h by E1A contributes to anticancer activity. Cancer Res.

[21] Su CM (2016). Mir-520h is crucial for DAPK2 regulation and breast cancer progression. Oncogene.

[22] Zhang M (2014). miR-302b is a potential molecular marker of esophageal squamous cell carcinoma and functions as a tumor suppressor by targeting ErbB4. J Exp Clin Cancer Res.

[23] Wu G (2014). miR-372 down-regulates the oncogene ATAD2 to influence hepatocellular carcinoma proliferation and metastasis. BMC Cancer.

[24] Tian RQ (2011). MicroRNA-372 is down-regulated and targets cyclin-dependent kinase 2 (CDK2) and cyclin A1 in human cervical cancer, which may contribute to tumorigenesis. J Biol Chem.

[25] Hu N (2011). miR-520b regulates migration of breast cancer cells by targeting hepatitis B X-interacting protein and interleukin-8. J Biol Chem.

[26] Zhang W, Kong G, Zhang J, Wang T, Ye L, Zhang X (2012). MicroRNA-520b inhibits growth of hepatoma cells by targeting MEKK2 and cyclin D1. PLoS One.

[27] Zhang S, Shan C, Kong G, Du Y, Ye L, Zhang X (2012). MicroRNA-520e suppresses growth of hepatoma cells by targeting the NF-kappaB-inducing kinase (NIK). Oncogene.

[28] Yan GJ (2014). MicroRNA miR-302 inhibits the tumorigenicity of endometrial cancer cells by suppression of Cyclin D1 and CDK1. Cancer Lett.

[29] Wang F (2012). hsa-miR-520h downregulates ABCG2 in pancreatic cancer cells to inhibit migration, invasion, and side populations. Br J Cancer.

[30] Banyard J, Bielenberg DR (2015). The role of EMT and MET in cancer dissemination. Connect Tissue Res.

[31] Chiu CC (2013). Grp78 as a therapeutic target for refractory head-neck cancer with CD24(−)CD44(+) stemness phenotype. Cancer Gene Ther.

[32] Li YC (2015). Areca nut contributes to oral malignancy through facilitating the conversion of cancer stem cells. Mol Carcinog.

[33] Lin SL, Chang DC, Ying SY, Leu D, Wu DT (2010). MicroRNA miR-302 inhibits the tumorigenecity of human pluripotent stem cells by coordinate suppression of the CDK2 and CDK4/6 cell cycle pathways. Cancer Res.

[34] Lin SL, Chang DC, Lin CH, Ying SY, Leu D, Wu DT (2011). Regulation of somatic cell reprogramming through inducible mir-302 expression. Nucleic Acids Res.

[35] Liu B, Li J, Cairns MJ (2014). Identifying miRNAs, targets and functions. Brief Bioinform.

[36] Xi Z, Huang R, Deng Y, He N (2014). Progress in selection and biomedical applications of aptamers. J Biomed Nanotechnol.

[37] Tessitore A (2015). Therapeutic Use of MicroRNAs in Cancer. Anticancer Agents Med Chem.

[38] Simonson B, Das S (2015). MicroRNA Therapeutics: the Next Magic Bullet?. Mini Rev Med Chem.

[39] Bouchie A (2013). First microRNA mimic enters clinic. Nat Biotechnol.

[40] Lu YC (2012). Oncogenic Function and Early Detection Potential of miRNA-10b in Oral Cancer as Identified by microRNA Profiling. Cancer Prev Res.

[41] Lu YC (2014). OncomiR-196 promotes an invasive phenotype in oral cancer through the NME4-JNK-TIMP1-MMP signaling pathway. Mol Cancer.

[42] Chang JT (2010). Highly potent and specific siRNAs against E6 or E7 genes of HPV16- or HPV18-infected cervical cancers. Cancer Gene Ther.

[43] Chen YJ (2013). DSG3 facilitates cancer cell growth and invasion through the DSG3-plakoglobin-TCF/LEF-Myc/cyclin D1/MMP signaling pathway. PLoS One.

[44] Chen CY (2015). Targeting annexin A2 reduces tumorigenesisi and therapeutic resistance of nasopharyngeal carcinoma. Oncotarget.

[45] Lin TY (2010). Proteomics of the radioresistant phenotype in head-and-neck cancer: Gp96 as a novel prediction marker and sensitizing target for radiotherapy. Int J Radiat Oncol Biol Phys.

[46] Lu YC (2015). Combined determination of circulating miR-196a and miR-196b levels produces high sensitivity and specificity for early detection of oral cancer. Clin Biochem.

[47] Lee LY (2015). Fascin is a circulating tumor marker for head and neck cancer as determined by a proteomic analysis of interstitial fluid from the tumor microenvironment. Clin Chem Lab Med.

