# Supplementary material for: MiR-520b as a novel molecular target for suppressing stemness phenotype of head-neck cancer by inhibiting CD44
Source: Sci Rep. 2017 May 17;7:2042. doi: 10.1038/s41598-017-02058-8 (PMC5435724; doi:10.1038/s41598-017-02058-8)
Supplement: Supplementary file 1 — Supplementary Information [file 41598_2017_2058_MOESM1_ESM.doc]

**MiR-520b as a novel molecular target for suppressing stemness phenotype of head-neck cancer by inhibiting CD44**

Ya-Ching Lu^1^, Ann-Joy Cheng^1,2#^, Li-Yu Lee^3^, Guo-Rung You^1^, Yan-Liang Li ^1^, Hsin-Ying Chen^1^, Joseph T Chang^2,4^*

^1^Department of Medical Biotechnology and Lab Science, College of Medicine, Chang Gung University, Taoyuan 333, Taiwan

^2^Department of Radiation Oncology, Chang Gung Memorial Hospital - Linko, Taoyuan 333, Taiwan

^3^Department of Pathology, Chang Gung Memorial Hospital - Linko, Taoyuan 333, Taiwan

^4^Department of Radiation Oncology, Xiamen Chang Gung Memorial Hospital, Xiamen, Fujian, China

**^#^Co-first author, equal contribution to the first author**

***Corresponding author**

Joseph T Chang, Professor

Department of Radiation Oncology

Chang Gung Memorial Hospital - Linko

E-mail: [cgmhnog@gmail.com](mailto:cgmhnog@gmail.com)

**Running Title:** MiR-520b inhibits cancer stemness

**Key words:** miR-520b, cancer stemness, CD44, head and neck cancer, therapeutic resistance

**Co-authors’ email addresses**

Ya-Ching Lu [achin515@gmail.com](mailto:achin515@gmail.com)

Ann-Joy Cheng [annjoycheng@gmail.com](mailto:annjoycheng@gmail.com)

Li-Yu Lee [r22068@adm.cgmh.org.tw](mailto:r22068@adm.cgmh.org.tw)

Guo-Rung You [guo.r.you@gmail.com](mailto:guo.r.you@gmail.com)

Yan-Liang Li [bluevancent121@yahoo.com.tw](mailto:bluevancent121@yahoo.com.tw)

Hsin-Ying Chen [chen.hsinying@gmail.com](mailto:chen.hsinying@gmail.com)

**Supplement Figure**

**Figure S1.** High level of CD44 was associated with shorter relapse-specific survival in HNC. The PrognoScan bioinformatic tool was used to analyze dataset GSE2837 in HNC patients (HR=1.25, *p*=0.021 with Kaplan-Meier analysis).


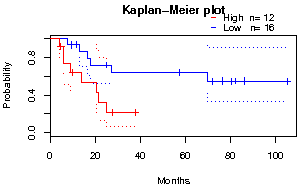


**Figure S2.** Lower level of miR-520b was associated with shorter overall survival in breast cancer. The bioinformatic tool of Kaplan-Meier Plotter was used to analyze TCGA dataset (HR=0.55, *P* = 0.023 with log rank analysis).


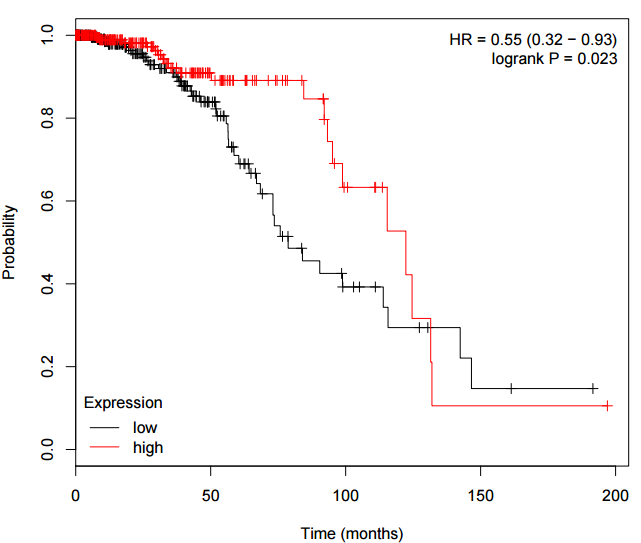


**Supplement**

**Supplemental Table 1:** The primary antibody used in this study

| **Name** | **Source** |
| --- | --- |
| CD44 | AM-1901b, Abgent, San Diego, CA, USA |
| E-cadherin  r-catenin  N-cadherin  Fibronectin | SC-7870, Santa Cruz Biotech, Santa Cruz, CA, USA  SC-8415, Santa Cruz Biotech, Santa Cruz, CA, USA  BD-610921, BD Biosciences, Franklin Lakes, NJ, USA  SC-18825, Santa Cruz Biotech, Santa Cruz, CA, USA |
| Nestin | SC-71665, Santa Cruz Biotech, Santa Cruz, CA, USA |
| Twist  Nanog  OCT4  Actin  GAPDH | SC-15393, Santa Cruz Biotech, Santa Cruz, CA, USA  SC-30331, Santa Cruz Biotech, Santa Cruz, CA, USA  #2750, Cell signaling, Danvers, MA, USA  MAB1502, Chemicon, Dublin, Rep of Ireland  SC-20357, Santa Cruz Biotech, Santa Cruz, CA, USA |
